# Genetic Diversity and Core Collection Construction of *Cymbidium ensifolium* var. susin

**DOI:** 10.3390/plants15091349

**Published:** 2026-04-28

**Authors:** Li Zhang, Tie Zhou, Yuxia Zhou, Yingshu Peng, Guolin Huang, Guimei Tang, Yang Liu, Yuanzhi Xiao, Fan Zhao, Weidong Li, Jilong Yang, Hongyan Fu

**Affiliations:** 1 Hunan Horticulture Research Institute, Hunan Academy of Agricultural Sciences, Changsha 410125, China; zhli911@hunaas.cn (L.Z.); 17873550809@163.com (T.Z.); yuxiaz75@163.com (Y.Z.); yspeng@hunaas.cn (Y.P.); huangguolin2002@hunaas.cn (G.H.); tangguimei@hunaas.cn (G.T.); liuyang@hunaas.cn (Y.L.); xiaoyuanzhi458@hunaas.cn (Y.X.); zhaofan2023@hunaas.cn (F.Z.); 2 Yuelu Shan Laboratory, Changsha 410125, China; 3 Hunan Key Laboratory of Germplasm Innovation and Comprehensive Utilization of Garden Flowers, Changsha 410125, China

**Keywords:** *Cymbidium ensifolium*, phenotypic traits, Hyper-seq sequencing, genetic diversity, core collection

## Abstract

Wild orchid populations are declining with intensified habitat fragmentation posing severe challenges to germplasm conservation. As an important ornamental Orchidaceae species, *Cymbidium ensifolium* has abundant germplasm resources and frequent natural and artificial hybridization. Long-term natural evolution and anthropogenic disturbance have led to complex genetic backgrounds and ambiguous phylogenetic relationships hindering accurate germplasm identification, elite resource excavation, and selective breeding. As a distinctive variety, *Cymbidium ensifolium* var. susin has great breeding potential. Clarifying its phenotypic and genetic characteristics is crucial for accelerating breeding progress. In this study, phenotypic determination, Hyper-seq reduced-representation genome sequencing, SNP/InDel genotyping, genetic diversity analysis, and core collection construction were used to evaluate the genetic diversity, population differentiation, and core germplasm screening of 13 *Cymbidium ensifolium* var. susin accessions. The results showed significant phenotypic differences and rich genetic variation among tested materials. Based on highly weighted floral traits, accessions were divided into three major phenotypic groups. At the molecular level, 963,239 SNP and 182,399 InDel loci were identified and mainly distributed in intergenic regions, followed by introns and exons. A phylogenetic tree was constructed from SNP loci combined with principal component and phenotypic clustering analyses. This study preliminarily clarified the genetic structure of pure-heart *Cymbidium ensifolium* var. susin, showing a distinct geographical pattern: “high consistency in Fujian and Guangdong; strong differentiation in Southwest China; and a transitional gradient in Central China”. Meanwhile, six core germplasm accessions were screened in this study, which provides a solid theoretical basis and material support for the conservation of pure-heart *Cymbidium ensifolium* var. susin accessions, variety improvement, hybrid parent selection, and molecular marker-assisted breeding. This is of great significance for promoting the innovation of Chinese orchid germplasm resources and the high-quality development of the industry.

## 1. Introduction

*Cymbidium ensifolium* belongs to the genus *Cymbidium* in the Orchidaceae family and is an important group of national orchids in China. It is also known as the “four-season orchid” due to its long flowering period and multiple flowering events. It can grow wild in mountain forests and also adapt well to artificially cultivated environments [1]. With elegant plant forms, rich floral fragrance, diverse flower patterns, and distinctive leaf esthetics, *Cymbidium ensifolium* is highly ornamental [2,3]. As a precious type of *Cymbidium ensifolium*, *Cymbidium ensifolium* var. susin has extremely high esthetic and economic value, serving as a core material for the breeding and industrial development of *Cymbidium ensifolium*. With the advancement of horticultural technology, the cultivation and propagation levels of *Cymbidium ensifolium* var. susin have been significantly improved, and its germplasm resources have become increasingly abundant. Germplasm resources are the foundation and key to variety improvement, innovation, and new variety breeding. Phenotypic trait identification is the most intuitive and traditional method for studying germplasm resource diversity [4]. Currently, this method has been widely applied to resource evaluation in crops [5,6,7], fruit trees [8,9,10], vegetables [11,12,13], ornamental plants [14,15,16], and other groups. Systematic analysis of the phenotypic diversity of *Cymbidium ensifolium* var. susin is of great significance for the exploration of excellent resources, germplasm innovation, and breeding utilization.

At present, systematic research on genetic resources remains relatively insufficient for the utilization of *Cymbidium ensifolium* var. susin germplasm. Genetic diversity, the core of species diversity, reflects differences in the gene pools and genetic structures of species [17], and serves as an important theoretical basis for the selective breeding of new varieties. Molecular markers, which directly reveal genetic variations at the genomic level, have the advantages of high accuracy, rich polymorphism, and independence from environmental factors and developmental stages [18,19,20], and have become key technologies for analyzing genetic diversity, population structure, and genetic relationships. Markers such as AFLP [21,22], RAPD [23,24], SRAP [25,26], SSR [27,28], ISSR [29,30], and SNP [31,32] have been widely used in orchid species identification and genetic evaluation. In recent years, Hyper-seq sequencing technology, characterized by low cost, high throughput, and high-density SNP genotyping, has been successfully applied to many plants, including those in the Orchidaceae [33], Cannaceae [34], and potato [35], providing an efficient tool for genetic diversity research.

Core germplasm refers to a representative germplasm subset that maximally preserves the genetic diversity, core genotypes and superior phenotypes of the entire germplasm population with a minimal sample size. Its construction is an effective way to simplify germplasm conservation procedures, reduce conservation costs, and boost the innovative utilization of elite germplasms. Morphological markers reflect genetic variations related to external phenotypic development, while molecular markers reveal genomic genetic differences within germplasms. Reliance solely on morphological traits or molecular data for core germplasm construction makes it difficult to fully and accurately reflect the overall genetic profile of germplasm resources. Integrating morphological traits and molecular data to construct core germplasms enables the dual characterization of phenotypic traits and genetic essence, maximizes the retention of genetic variation in original germplasms, and significantly improves the representativeness, scientificity and practicality of core germplasms. Significant habitat heterogeneity exists across different geographical regions. Long-term geographical isolation and adaptive evolution have shaped unique genetic characteristics of *Cymbidium ensifolium* populations. Studies based on a single geographical provenance cannot reflect its overall genetic diversity, evolutionary patterns, and regulatory mechanisms of geographical environments on genetic differentiation, and thus fail to ensure the scientificity of core germplasm screening. Therefore, selecting *Cymbidium ensifolium* germplasms from different geographical origins to conduct research on population genetic structure, phylogenetic relationships and biogeography is more scientific and comprehensive.

Against the aforementioned research background and requirements, 13 accessions of *Cymbidium ensifolium* var. *susin* from different geographical origins were employed as research materials in this study. Phenotypic traits were systematically analyzed, and SNP markers were developed based on Hyper-seq sequencing to explore genetic variation. Furthermore, genetic relatedness and population genetic structure were analyzed, and core germplasms were finally constructed. This study aims to provide a scientific basis for the conservation, evaluation and innovative utilization of *Cymbidium ensifolium* var. susin accessions.

## 2. Materials and Methods

### 2.1. Plant Material

A total of 13 *Cymbidium ensifolium* var. susin accessions collected from Fujian, Guangdong, Hunan and Sichuan provinces were planted in the Orchid Resource Garden of Hunan Provincial Horticultural Research Institute. Detailed information on these 13 germplasm materials is presented in Table 1.

### 2.2. Measurement of Phenotypic Traits

The selection of phenotypic traits is mainly based on the “Guidelines for the conduct of tests for distinctness, uniformity and stability Cymbidium Sw. NY/T 2441-2013” [36], combined with correlation analyses of phenotypic traits in *Cymbidium ensifolium* [37,38,39]. A total of 30 phenotypic traits were selected for statistics, of which 20 were quantitative traits and 10 were quality traits. Quantitative traits were measured in centimeters. All indices were measured in triplicate, and the average value was calculated. The measurement methods for quantitative traits and assignment rules for quality traits are detailed in Table 2.

### 2.3. Library Construction and Sequencing

Library construction and sequencing were commissioned to BeneGene, and the sequencing was performed on the Illumina NovaSeq 6000 platform (second-generation sequencing technology). The Hyper-seq sequencing method was adopted to simplify the genome sequencing process. After sequencing, the offline raw data were subjected to quality control and filtering using fastp software (version: 0.23.4; parameters: default settings) [40]. Subsequently, the filtered reads were aligned to the Jianlan reference genome using bwa software (version: 0.7.17; parameters: mem) [41].

### 2.4. Variant Identification

Variant detection was performed on all samples using GATK (version 4.4.0.0) with the HaplotypeCaller, CombineGVCFs, and GenotypeGVCFs modules. To minimize false-positive variants, the identified SNPs and InDels were filtered following the official GATK hard-filtering recommendations using GATK (version 4.2.5.0) with the VariantFiltration tool. The detected SNPs and InDels were subsequently annotated using ANNOVAR (version 20200607) with the convert2annovar.pl and annotate_variation.pl scripts.

### 2.5. Population Phylogenetic Tree Construction

The neighbor-joining (NJ) method in PHYLIP (version 3.696, module: neighbor) was used to construct the phylogenetic tree. Subsequently, the resulting tree in Newick format was visualized using the ggtree package.

### 2.6. Population Principal Component Analysis and Genetic Structure Analysis

Principal component analysis (PCA) was performed using GCTA (version 1.93.2) with the parameters -grm and -pca [42]. Prior to PCA, quality control of variant sites was conducted using VCFtools (version 0.1.17) with the parameters -maf, -max-missing, -min-alleles, -max-alleles, and remove-indels. Specifically, variant sites with a minor allele frequency (MAF) less than 0.05 and a genotype missing rate greater than 20% were excluded, retaining only single nucleotide polymorphism (SNP) sites with minor alleles. Population genetic structure analysis was performed based on the filtered SNP sites using ADMIXTURE (version 1.3.0) with the parameter -cv inputFile K. The K values were set from 2 to 10, and the optimal K value was determined according to the cross-validation error rate.

### 2.7. Evaluation of Core Germplasm Screening

Core germplasm was screened using Genocore V2.0. Principal component analysis (PCA) was employed to compare the original germplasm materials with the screened core germplasm materials for evaluation purposes. This comparison aimed to verify the representativeness of the core germplasm, ensuring that it could effectively capture the genetic variation in the original germplasm population. Core collection screening was performed based on maximum genotype coverage combined with an appropriate sampling proportion. Calculated routine genetic diversity parameters, including polymorphic loci, effective number of alleles (*Ne*), Shannon-Wiener diversity index (*H*′), Nei’s gene diversity index, and polymorphism information content (PIC), to evaluate the genetic diversity of original germplasms and screened core germplasms.

### 2.8. Statistical Analysis

Statistical analysis was performed using Excel 2021 (Microsoft, USA) to calculate the mean, maximum, minimum, standard deviation (SD), coefficient of variation (CV), and Shannon–Wiener genetic diversity index (*H*′) for phenotypic traits. The coefficient of variation (CV), a normalized measure of relative dispersion in a probability distribution, was calculated as: CV = (SD/Mean) × 100%. The genetic diversity index was computed as *H*′ = −∑PilnPi where Pi represents the proportion of accessions in the i-th class of a given phenotypic trait relative to the total number of accessions. Cluster analysis of phenotypic quantitative traits was performed via hierarchical clustering with Ward’s minimum variance method based on Euclidean distance. The phenotypic dendrogram was constructed to classify all tested materials into distinct phenotypic groups. Correlation analysis, principal component analysis (PCA), and cluster analysis were conducted using Origin 2022 (OriginLab, USA), and the corresponding figures were plotted.

## 3. Results

### 3.1. Phenotypic Trait Analysis of Cymbidium ensifolium var. susin

#### 3.1.1. Analysis of Genetic Diversity Based on Phenotypic Traits

In this study, the genetic diversity of phenotypic traits was analyzed in 13 germplasms of *Cymbidium ensifolium* var. susin. Phenotypic data (Table 3 and Table 4) showed that the coefficient of variation (CV) of quantitative traits ranged from 6.66% to 36.74%, and the average of CV was 13.69%. The genetic diversity index (H′) of quantitative traits varied from 0.54 to 1.93, and the mean value of H′ was 1.51. The highest H′ value observed for flower length (FL) suggests abundant genetic diversity in this trait among the tested germplasms. In contrast, the lowest H′value for petal color number (PCN) indicates that this trait is relatively stable and exhibits little variation across different germplasms. The results of the frequency distribution of qualitative traits showed that the frequency distribution of qualitative traits in different distribution regions was unbalanced. The H′ values of qualitative traits ranged from 0.54 to 1.27. Among them, petal posture (PP) exhibited the highest H′value, followed by median sepal shape (MSS) and blade shape (BS), plant type (PT), petal shape (PS), lateral sepal shape (LSS), and twisted leaves (TL) showed the lowest H′ values.

#### 3.1.2. Principal Component Analysis Based on Phenotypic Traits

**Figure 1:**
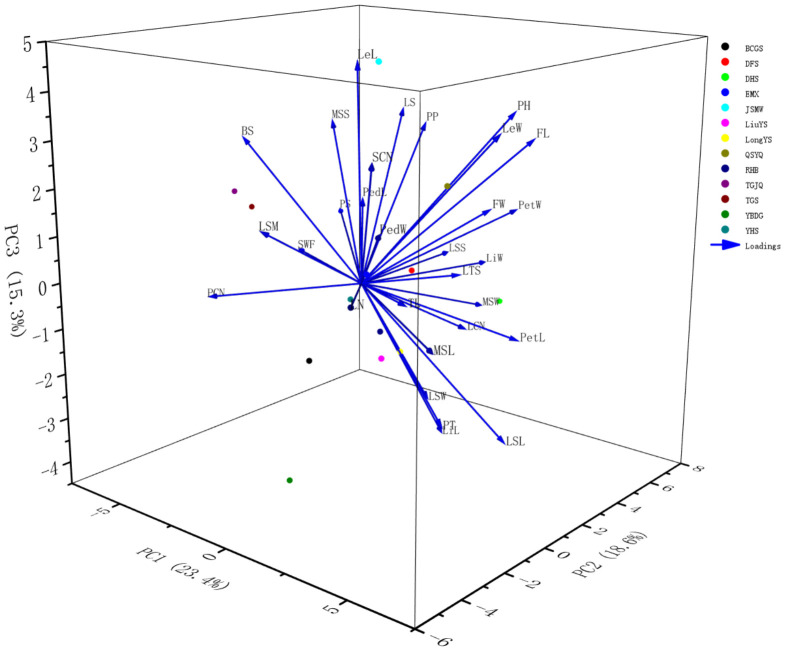
Principal component analysis diagram of *Cymbidium ensifolium* var. susin. Note: Each colored dot represents an individual accession, with colors corresponding to the legend. Blue arrows indicate the principal component loadings of the morphological traits.

Principal component analysis (PCA) was performed to comprehensively evaluate 30 phenotypic traits of 13 *Cymbidium ensifolium* var. susin accessions (Table 5, Figure 1). Using the criterion of eigenvalue > 1, a total of 8 principal components were extracted, with a cumulative variance contribution rate of 93.89%, which could effectively reflect the overall information of phenotypic traits of the tested germplasms. The first principal component accounted for 23.38% of the total variance, with high loadings on MSL, LSL and PetL, mainly reflecting the length characteristics of floral organs. The second principal component explained 18.58% of the variance, with high loadings on LiW, PetW and MSW, mainly representing the width characteristics of floral organs. The variance contribution rates of the third to eighth principal components were 15.34%, 9.95%, 9.54%, 6.67%, 6.12% and 4.30%, respectively, with high loadings on LeL, LTS, LSW, TL, LSM and PedL, which mainly reflected leaf length, leaf shape, leaf surface markings and pedicel characteristics.

Comprehensive PCA results indicated that quantitative traits of floral parts dominated the phenotypic variation of 13 *Cymbidium ensifolium* var. susin accessions, serving as key phenotypic identification indicators.

#### 3.1.3. Correlation Analysis of Phenotypic Traits

**Figure 2:**
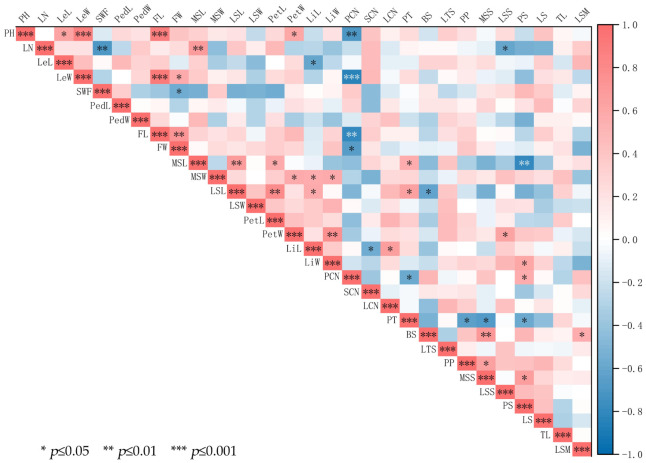
Correlation coefficients of phenotypic traits in *Cymbidium ensifolium* var. susin.

There are different degrees of correlation among the 13 *Cymbidium ensifolium* var. susin accessions (Figure 2). Among the 40 pairs of phenotypic traits, 25 pairs exhibited positive correlations, while the remaining 15 pairs showed negative correlations. Specifically, leaf width (LeW) and plant height (PH), leaf width (LeW) and flower length (FL), as well as flower length (FL) and plant height (PH), displayed extremely significant positive correlations with correlation coefficients exceeding 0.88. Additionally, extremely significant positive correlations were observed between petal width (PetW) and lip width (LiW), median sepal length (MSL) and leaf number (LN), median sepal length (MSL) and lateral sepal length (LSL), lateral sepal length (LSL) and petal length (PetL), and blade shape (BS) and median sepal shape (MSS). In contrast, petal color type number (PCN) was extremely significantly negatively correlated with leaf width (LeW); meanwhile, extremely significant negative correlations were also found between petal color type number (PCN) and flower length (FL), petal color type number (PCN) and plant height (PH), scape with flowers (SWF) and leaf number (LN), as well as petal shape (PS) and median sepal length (MSL). The above results indicate that there are significant or highly significant correlations among most floral traits of 13 *Cymbidium ensifolium* var. susin accessions.

#### 3.1.4. Clustering Analysis of Phenotypic Traits

**Figure 3:**
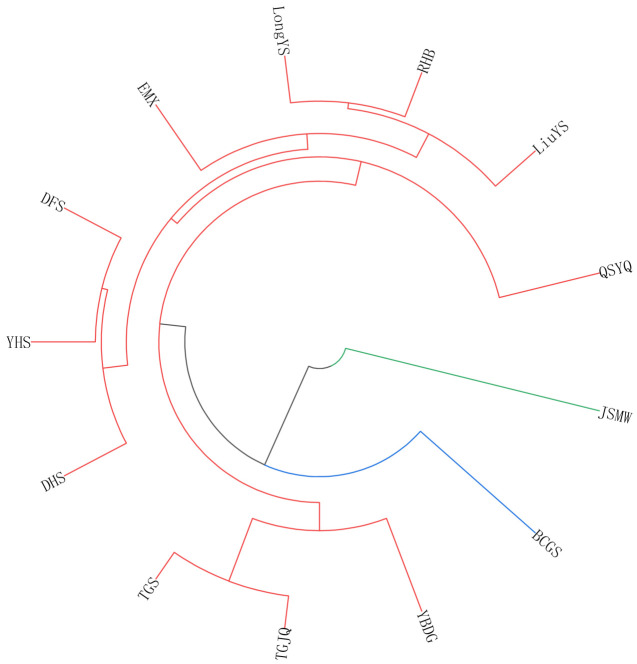
Phenotypic trait clustering analysis of *Cymbidium ensifolium* var. susin. Note: Different colored branches represent distinct genetic clusters: green, Cluster 1; blue, Cluster 2; red, Cluster 3.

Based on phenotypic traits, the 13 *Cymbidium ensifolium* var. susin accessions were clustered into three groups (Figure 3). Group I consisted solely of JSMW, which was mainly characterized by taller plant height, longer leaf length, longer pedicel length, narrower flower width, and shorter lip length. Group II contained only BCGS, with the primary phenotypic features of fewer leaves, shorter leaf length, and narrower pedicel width. Group III comprised 11 germplasm accessions, namely QSYQ, LiuYS, TGS, TGJQ, DFS, DHS, RHB, LongYS, YHS, YBDG, and EMX, whose core phenotypic characteristics included longer and wider lip, longer and wider petals, and wider median sepal. These findings were consistent with those obtained from principal component analysis.

#### 3.1.5. Analysis of Floral Phenotypic Traits

**Figure 4:**
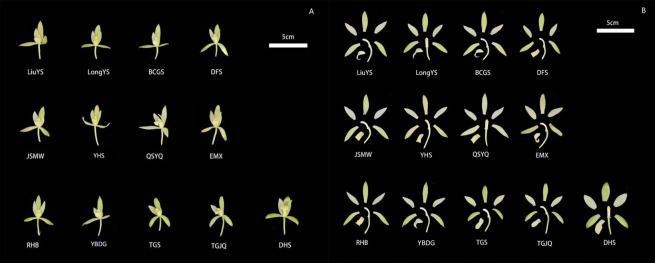
Floral morphology and anatomical diagram of *Cymbidium ensifolium* var. susin. Note: (**A**): Intact flowers showing the overall floral morphology; (**B**): Dissected flowers showing the anatomical structures of floral organs (sepals, petals, labellum, and column). Bar = 5 cm.

Morphological and anatomical observations were conducted on 13 *Cymbidium ensifolium* var. susin accessions at the full-bloom stage, to compare and analyze the differentiation characteristics of floral external morphology and internal structure among different accessions (Figure 4). The results showed that all tested materials produced typical actinomorphic flowers of Orchidaceae under natural growth conditions, with highly uniform overall size of floral organs. Flower color was predominantly pale yellow-green or white-green, which is a typical diagnostic trait that distinguishes *Cymbidium ensifolium* var. susin from common *Cymbidium ensifolium* groups. Phenotypic differences among accessions were mainly reflected in sepal and petal morphology, as well as floral architecture: most accessions (e.g., LiuYS, LongYS, BCGS, RHB, DFS, JSMW, YBDG) exhibited upright and compact flower shapes with flat, stretched, and regular perianths; several accessions (e.g., YHS, QSYQ, EMX, TGS, TGJQ) showed obvious distortion and curling of sepals and petals, with prominent phenotypic variation; and a small number of accessions (e.g., DHS) possessed broader petals and higher floral fullness.

### 3.2. Hyper-Seq Sequencing and Population Evolutionary Analysis

#### 3.2.1. Quality Control and Comparative Analysis of Genome Sequencing Data

The Hyper-seq simplified genome sequencing method was employed to sequence 13 *Cymbidium ensifolium* var. susin accessions. The statistical results are presented in Table 6. A total of 205,823,776 raw reads were generated, and 205,708,984 high-quality clean reads were obtained after filtering, with an average of 15,823,768 clean reads per germplasm. Among them, TGJQ had the maximum number of clean reads (22,337,560), whereas YBDG had the minimum (9,414,972). The average values of sequencing quality Q20 and Q30 were 96.00% and 90.07%, respectively. The GC content ranged from 38.67% to 40.76%, with an average of 39.54%. The clean reads were aligned to the *Cymbidium ensifolium* reference genome [41].

#### 3.2.2. Statistics and Distribution of Nucleotide Variations

**Figure 5:**
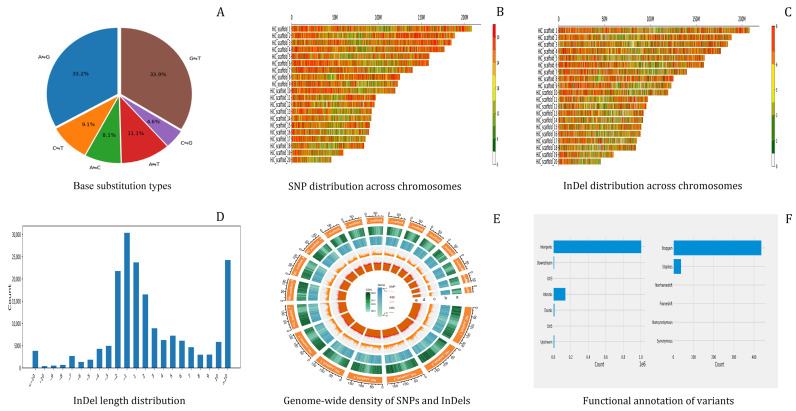
Genome-wide analysis of SNP and InDel variants in *Cymbidium ensifolium* var. susin. Note: (**A**): Base substitution types; (**B**): SNP distribution across chromosomes; (**C**): InDel distribution across chromosomes; (**D**): InDel length distribution; (**E**): Genome-wide density of SNPs and InDels; (**F**): Functional annotation of variants.

To elucidate the core genetic variation characteristics among 13 *Cymbidium ensifolium* var. susin accessions, a total of 1,145,638 biallelic variants were identified by mapping to the reference genome. The numbers of triallelic, tetraallelic, pentaallelic, hexaallelic, and heptaallelic variants were 34,378, 1720, 115, 31, and 21, respectively. Analysis of base substitution types across all variant loci revealed that A→G transition and G→T transversion events were the predominant mutation types, accounting for 33.9% and 33.2% of the total variations, respectively (Figure 5A).

Chromosomal mapping of the 963,239 SNP population loci demonstrated significant disparities in SNP distribution among different chromosomes, as well as uneven distribution across different regions within the same chromosome. Specifically, chromosome 1 harbored the highest number of SNP variants (53,053), while chromosome 20 contained the fewest (only 9405; Figure 5B), with a genome-wide average of 48,161.95 SNPs per chromosome.

Statistical analysis of population InDel variations was performed on a per-chromosome basis (Figure 5C). The results showed that the 182,399 InDel variants exhibited remarkable differences in abundance among chromosomes and uneven distribution within each chromosome. Chromosome 1 possessed the largest number of InDel variants (11,496), whereas chromosome 20 had the smallest (only 2081), with a genome-wide average of 9119.95 InDel variants per chromosome.

InDel lengths were quantified with positive values representing deletions and negative values representing insertions. The results indicated that InDel variations were dominated by deletion events (Figure 5D). The three most abundant variant types, in descending order, were single-base insertions, deletions of more than 10 bases, and single-base deletions.

Analysis of genome-wide variation distribution patterns revealed that both SNP and InDel variants displayed significant regional heterogeneity across the genome (Figure 5E). SNP density was markedly higher than InDel density, and both variant types were enriched in intergenic regions and introns. In contrast, variant densities were relatively low in euchromatic regions with high gene density, reflecting the high evolutionary conservation of coding regions.

Functional annotation of the identified SNP and InDel variants was conducted to identify genes affected by variations and evaluate the potential impacts of mutations on gene function (Figure 5F). The results showed that SNP and InDel variants were mainly distributed in intergenic regions, followed by introns and exons. Variations in exonic regions could directly alter the base composition and arrangement of coding sequences, thereby causing changes in gene structure. Based on variant types and their effects on gene function, exonic variants were further classified into synonymous mutations, missense mutations, frameshift deletions, frameshift insertions, stop-gain mutations, and stop-loss mutations. Among exonic SNPs, non-synonymous single nucleotide variants (SNVs) accounted for 51.19%, synonymous SNVs for 29.18%, frameshift deletions for 1.02%, and frameshift insertions for 7.92%.

#### 3.2.3. Phylogenetic Tree Construction and Population Principal Component Analysis

**Figure 6:**
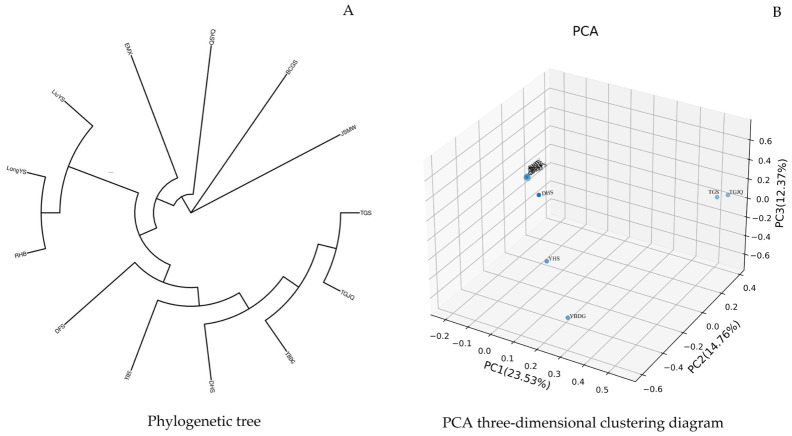
Combined chart of phylogenetic tree and principal component analysis of *Cymbidium ensifolium* var. susin. Note: (**A**): Phylogenetic tree; (**B**): PCA three-dimensional clustering diagram.

Branch lengths in the phylogenetic tree intuitively reflect the genetic distance among germplasms. Longer branches indicate richer accumulation of genetic variation and higher differentiation between germplasms. Nodes represent the most recent common ancestor of the tested materials; nodes closer to the branch tips suggest closer genetic relationships and later divergence times among accessions. Based on the screened 963,239 high-quality SNP loci, a phylogenetic tree was constructed in this study (Figure 6A). The results showed that the 13 *Cymbidium ensifolium* var. susin accessions were clustered into three clades. Cluster I comprised three accessions: LongYS, RHB, and LiuYS, which formed a tight monophyletic clade with extremely short branch lengths, indicating an extremely close genetic distance and highly consistent genetic background among them. Cluster II contained six accessions: DFS, YHS, DHS, YBDG, TGJQ, and TGS, presenting a continuously nested clustering structure with gradual genetic differentiation and high homology in their genetic background within the cluster. Cluster III consisted of four accessions: EMX, QSYQ, BCGS, and JSMW, all of which exhibited independent long branches, showing the farthest genetic distance from the other clusters and the most pronounced genetic specificity.

Principal component analysis was performed on the 13 *Cymbidium ensifolium* var. susin accessions based on 963,239 SNP loci. The results revealed that the first three principal components (PC1, PC2, and PC3) explained 23.53%, 14.76%, and 12.37% of the total variance, respectively, with a cumulative contribution rate of 50.66%. In the PCA scatter plot (Figure 6B), the 13 *Cymbidium ensifolium* var. susin accessions were clearly divided into three genetic groups. Group I included nine accessions: LongYS, RHB, LiuYS, DFS, EMX, QSYQ, BCGS, JSMW, and DHS, which were highly concentrated in distribution, indicating highly homologous genetic backgrounds. Group II was composed of two accessions, TGS and TGJQ, which were distinctly separated from Group I along the PC1 axis. Group III contained two accessions, YHS and YBDG, with high discreteness, representing an independent group with relatively distant genetic relationships.

#### 3.2.4. Population Structure Analysis Based on SNPs

**Figure 7:**
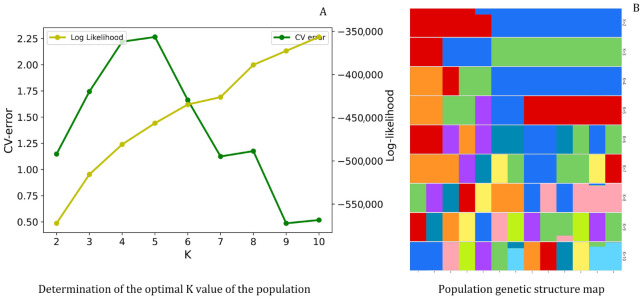
Population genetic structure analysis of *Cymbidium ensifolium* var. susin. Note: (**A**): Determination of the optimal K value for the population. Cross-validation error (CV error, green, left axis) and log-likelihood (yellow, right axis) across K values, the optimal K is selected at the minimum CV error; (**B**): Population genetic structure map. Each vertical bar represents one individual. Different colors denote distinct ancestral components, with their proportions indicating the corresponding genetic contribution.

Population structure analysis was performed on the 13 *Cymbidium ensifolium* var. susin accessions collection with the number of assumed subpopulations *(K*) set from 2 to 10. Cross-validation error (CV error) was calculated for each K to evaluate clustering performance. The analysis of the optimal *K* value for population genetic grouping is shown in Figure 7A. The cross-validation (CV) error curve showed a distinct inflection point and reached its peak at *K* = 5 and decreased to the global minimum at *K* = 9; the log-likelihood value showed an opposite trend to the CV error. Statistically, the model exhibited the optimal fitting performance at *K* = 9. However, the declining rate of CV error slowed down substantially when *K* > 5, and the improvement in fitting efficiency derived from subgroup subdivision was greatly weakened. This indicates that the grouping at *K* = 9 presents obvious overfitting, which excessively splits genetic components and lacks reliable biological support. As a critical inflection point of the curve, *K* = 5 acts as an important threshold for population genetic structure differentiation.

Cross-validation based on population genetic structure clustering (Figure 7B) and PCA results revealed that the tested population formed three genetic clusters with clear boundaries and stable differentiation at *K* = 3. The grouping results were highly consistent with the geographical origin, ecological distribution and core phenotypic traits of experimental materials, which could accurately reflect the fundamental genetic differentiation pattern shaped by long-term evolution and represent the core genetic divergence background of the population. When *K* ≥ 5, the population was further subdivided into subclusters on the basis of the original major groups. Such differentiation is mainly attributed to local genetic drift, small-scale gene flow and recent genetic admixture, belonging to secondary genetic variation. These subdivisions are weakly correlated with species macroevolution and geographical differentiation of germplasm resources, thereby having limited reference value.

In summary, combining cross-validation statistics, model fitting effectiveness, overfitting risk, and multiple biological factors including germplasm geographical origin, phenotypic differentiation and evolutionary background, *K* = 3 was determined as the optimal genetic grouping with the highest biological interpretability for the tested population.

#### 3.2.5. Screening and Evaluation of Genetic Diversity in Core Germplasm

Core germplasm aims to maximally represent the genetic diversity of the entire resource population including geographical distribution with the minimum number of genetic resources, so as to reduce the cost of germplasm conservation. Genotype coverage refers to the proportion of all genotypes detected in the entire germplasm collection that can be covered by the core germplasm, which reflects the representativeness of the core germplasm. Sampling proportion refers to the proportion of the core germplasm in the entire germplasm collection, which balances the representativeness and economy of the core germplasm. In this study, samples were randomly selected from core and non-core germplasms at sampling ratios of 25%, 50%, 75% and 100% to statistically analyze multiple genetic parameters. The results showed that a total of 6 core germplasm accessions were screened under the 100% sampling ratio (Table 7). The proportion of polymorphic loci in the core germplasm reached 91.81%, which was significantly higher than that in the non-core germplasm population. The effective number of alleles (*Ne*) was 1.51, the Shannon–Wiener diversity index (*H*′) was 0.68, and Nei’s gene diversity index (*He*) was 0.61. Overall, no significant differences were observed between the core and non-core germplasm populations for these genetic diversity parameters.

**Figure 8:**
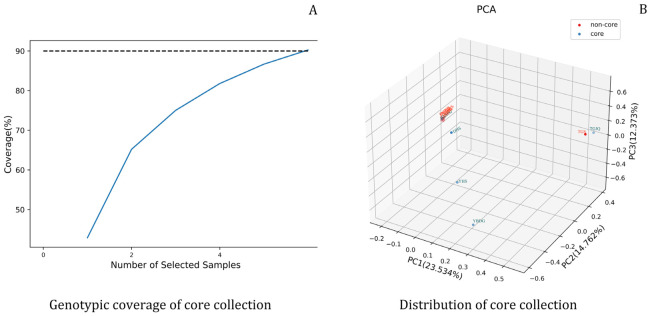
Genotypic coverage and distribution of the core collection of *Cymbidium ensifolium* var. susin. Note: (**A**): Genotypic coverage of core collection. The blue solid line shows the observed genotypic coverage of core alleles as the number of selected samples increases, the black dashed line indicates the predefined target coverage threshold of 90%; (**B**): Distribution of core collection. The red dots represent individuals from the non-core collection, and the blue dots represent individuals from the core collection.

To further verify the representativeness of the core germplasm, genotype coverage analysis was conducted for both core and non-core germplasm separately, and the distribution characteristics of the core germplasm in the total sample were evaluated (Figure 8A). The results showed that the 6 selected core germplasm accessions could cover 90.26% of the total genotypes, with rich genetic types. Meanwhile, the results of principal component analysis (Figure 8B) indicated that the core germplasm was relatively uniformly distributed among the preserved germplasm, which could well represent the genetic diversity of the original germplasm.

## 4. Discussion

### 4.1. Phenotypic Diversity Analysis of Cymbidium ensifolium var. susin

Phenotypic diversity is the core foundation for the evaluation and breeding utilization of germplasm resources, and its variation characteristics directly determine the breeding potential of germplasm materials. The coefficient of variation (CV) is a core indicator for quantifying the degree of trait variation; generally, CV > 10% is used as an important criterion for judging significant trait differences among germplasms [43]. The Shannon–Wiener diversity index (H′) is a key indicator for evaluating the richness and evenness of genetic diversity [44], with higher index values representing richer genetic diversity within the population. The results of this study showed that the CV of quantitative traits in the tested materials ranged from 6.66% to 36.74%, with an average CV of 13.69%. The H′ of quantitative traits varied from 0.54 to 1.93, while that of qualitative traits ranged from 0.54 to 1.27. Collectively, these results indicate that the tested germplasms exhibit significant differences in phenotypic traits and harbor abundant phenotypic variation within the population, providing a solid genetic basis for subsequent cross-breeding, elite individual selection, and other related studies. The study also found that the ranking orders of CV and H′ across traits were not completely consistent, which fundamentally arises from essential differences in the evaluation dimensions of the two indicators. CV primarily quantifies the degree of dispersion and magnitude of variation in trait values, whereas H′ mainly characterizes the distribution pattern of different phenotypic classes for a given trait, namely the richness and evenness of diversity. This phenomenon has been reported in phenotypic diversity studies of various plant species, including rose, lotus, Chinese chestnut, and sesame [45,46,47,48], representing a common pattern in plant phenotypic evaluation, which also indirectly confirms the scientificity and reliability of the phenotypic analysis results in this study.

Principal component analysis is a classic method for plant phenotypic evaluation that simplifies the evaluation system and identifies core traits while retaining key information [49]. In this study, eight principal components (cumulative contribution rate 93.89%) were extracted from 30 phenotypic traits via PCA, fully capturing core phenotypic information and eliminating redundant traits. Among them, quantitative floral traits (MSL, LSL, PetL, LiW, PetW, MSW) had high weights, serving as core traits reflecting phenotypic differences among germplasms and key indicators for new variety breeding, thus providing a reference for targeted hybrid parent selection. Correlation analysis clarifies internal trait relationships, optimizes breeding strategies, and improves breeding efficiency [50]. The results of this study showed that most floral traits were significantly or highly significantly correlated, consistent with findings in ornamental plants such as orchids, lotus, and roses [51,52,53], reflecting the universality of coordinated floral trait variation in ornamental plants. This enables the indirect selection of target traits via key indicator traits, thereby shortening the breeding cycle and improving precision. Cluster analysis enables scientific germplasm classification, providing a basis for germplasm management, genetic difference identification, and parental combination. 13 *Cymbidium ensifolium* var. susin accessions were divided into three clusters via cluster analysis, with inter-cluster differences mainly concentrated in quantitative floral traits. These results were mutually verified with PCA and consistent with phenotypic studies on floral traits in Pleione bulbocodioides, Tulipa gesneriana, and Phalaenopsis [54,55,56], further confirming the reliability of the analysis. Quantitative floral traits are key criteria for germplasm classification and parental selection, providing a reference for germplasm management and breeding practice. Furthermore, the significant differentiation in floral phenotypes intuitively reflects the rich morphological diversity of *Cymbidium ensifolium* var. susin accessions, which can provide an important phenotypic basis for the screening of excellent ornamental accessions, trait evaluation and subsequent genetic differentiation research.

In summary, systematic analysis of 30 phenotypic traits showed significant differences and high diversity among the 13 *Cymbidium ensifolium* var. susin accessions. PCA, correlation and cluster analyses identified key floral traits and three germplasm groups, highlighting the core role of floral traits in phenotypic evaluation and breeding. The results provide a theoretical basis for variety improvement, parental selection and breeding efficiency enhancement.

### 4.2. Hyper-Seq Sequencing and Population Evolutionary Analysis of Cymbidium ensifolium var. susin

At present, molecular markers have become the main approach for the analysis of genetic diversity and genetic relationships [57]. As the most stable and abundant type of genetic variation in most genomes, SNPs are widely used in the construction of high-density genetic maps, quantitative trait locus mapping, and genetic diversity analysis due to their high availability and high marker density, compared with traditional molecular markers [58]. In this study, the genetic variation and diversity of 13 *Cymbidium ensifolium* var. susin accessions were comprehensively analyzed via Hyper-seq sequencing. A total of 963,239 population SNP loci and 182,399 population InDel loci were identified. The mapping rates of these germplasms ranged from 98.90% to 99.36%, thereby confirming the high quality of the sequencing data as well as the close genetic relationship among the samples. Variant annotation analysis revealed that SNP and InDel variations mainly occurred in intergenic regions.

To clarify the genetic relationships among 13 *Cymbidium ensifolium* var. susin accessions, a phylogenetic tree and principal component analysis were conducted based on SNP data. Both methods consistently clustered all accessions into three groups, mutually validating the clustering reliability. Specifically, DHS and TGS in Group I were closely related; Group II included the TGS-TGJQ clade; YHS and YBDG in Group III were highly discrete in PCA, consistent with their early differentiation and unique genetic background in the phylogenetic tree. Combined with phenotypic clustering, the genetic-geographical pattern of this orchid was defined as “high consistency in Fujian-Guangdong, strong differentiation in Southwest China, and transitional variation in Central China”. Accessions from Nanjing (Fujian, including DFS, DHS, YHS, JSMW, QSYQ), Puning and Meizhou (Guangdong, including TGS, TGJQ, YBDG) formed a core clade with short branches and aggregated nodes, sharing a consistent genetic background. This supports that the Fujian-Guangdong border is an important distribution and genetic diversity center for Chinese orchids, where favorable climate and frequent gene flow reduced genetic differentiation. In contrast, accessions from Mount Emei (Sichuan, EMX) and Xiangxi (Hunan, BCGS) had the longest branches, diverging significantly from the core group with unique genetic backgrounds, due to adaptive evolution from long-term geographical isolation. Additionally, accessions from Longyan (Fujian, LongYS), Liuyang (Hunan, LiuYS) and Renhua (Guangdong, RHB) formed independent lineages with moderate branches, showing a Fujian-Hunan-Guangdong geographical gradient transition. In conclusion, significant geographical genetic differentiation of *Cymbidium ensifolium* var. susin exists in southern China, providing a valuable reference for elucidating the dispersal, evolution and germplasm differentiation of Chinese orchids.

The construction of core germplasm is a core strategy for the conservation and efficient utilization of plant genetic resources. It retains the genetic diversity of the original population with the minimum sample size, reduces the costs of conservation and identification, and improves breeding efficiency [59]. In this study, a total of 6 core germplasm accessions were selected. They were evenly distributed among the total samples, covering 90.26% of the genotypes of the entire population. Moreover, the average number of effective alleles, Shannon’s information index, and gene diversity index were all higher than those of the retained germplasms, which can fully present the genetic variation characteristics of the original population. This provides an efficient carrier for germplasm conservation, utilization, and parent selection, effectively saving human and material input.

The combined analysis of phenotypic and genetic diversity is the key to deciphering the genetic mechanisms of agronomic traits [60]. This study integrated phenotypic variation and molecular genetic information, clarified the phenotypic differentiation rules and genetic structure characteristics of 13 *Cymbidium ensifolium* var. susin accessions, and accurately located the key genes and genomic regions related to target traits. It provides scientific support for hybrid parent selection and directional trait improvement, effectively shortens the breeding cycle, improves breeding accuracy, and promotes the standardized and efficient development of genetic breeding work of *Cymbidium ensifolium* var. susin.

In this study, 13 *Cymbidium ensifolium* var. susin accessions were sampled from Fujian, Guangdong, Hunan and other key regions, covering the core natural distribution areas of *Cymbidium ensifolium* and exhibiting higher representativeness than those in previous studies. Nevertheless, the sampling coverage was still regionally restricted. Future research will expand the geographic sampling range and germplasm quantity to further clarify the population genetic background of *Cymbidium ensifolium* var. susin. For analytical strategies, this study integrated phenotypic principal component analysis, cluster analysis, molecular markers and population structure analysis. The joint comparison of phenotypic traits and molecular genetic data enabled mutual verification and complementary analysis, avoiding the limitations of a single evaluation method. This comprehensive analytical framework provides solid theoretical support for the precise identification and rational utilization of *Cymbidium ensifolium* var. susin accessions. Further research will adopt transcriptomic and metabolomic multi-omics approaches to mine key functional genes and elucidate the genetic differentiation patterns and adaptive evolution mechanisms of *Cymbidium ensifolium* var. susin accessions. In terms of breeding practice, core germplasms were screened through combined morphological and molecular marker analysis. This finding offers a reliable reference for germplasm conservation and genetic improvement, with superior practical applicability compared with single-index evaluation. Furthermore, the core germplasm bank will be continuously enriched and optimized to sustain genetic diversity, thereby underpinning the long-term preservation, sustainable utilization and breeding innovation of *Cymbidium ensifolium* var. susin accessions.

## 5. Conclusions

In this study, 13 *Cymbidium ensifolium* var. susin accessions were used as experimental materials. Phenotypic morphological identification and Hyper-seq reduced-representation genome sequencing were adopted to systematically evaluate 30 phenotypic traits and molecular genetic characteristics. The results showed that the CV of quantitative traits ranged from 6.66% to 36.74%, with an average of 13.69%. The H′ values of quantitative traits were 0.54–1.93, while those of qualitative traits were 0.54–1.27, indicating significant phenotypic differentiation and abundant genetic variation among accessions. Correlation analysis revealed significant or highly significant correlations among most floral traits. Combined with principal component analysis and cluster analysis, floral quantitative traits with high contribution weights were screened, and the 13 tested accessions were classified into three major groups. A total of 963,239 SNP loci and 182,399 InDel loci were identified across the populations. Variant annotation showed that SNP and InDel variants were mainly distributed in intergenic regions, followed by introns and exons. Phylogenetic tree construction and principal component analysis based on genome-wide SNP loci consistently divided the 13 *Cymbidium ensifolium* var. susin accessions into three clusters. Combined with phenotypic clustering, preliminary analysis indicated that the 13 *Cymbidium ensifolium* var. susin accessions presented high genetic consistency in Fujian and Guangdong, significant genetic differentiation in Southwest China, and transitional variation characteristics in Central China. A total of 6 core collections with an allele retention rate of 90.26% were screened, providing empirical data and scientific support for the efficient conservation, accurate identification and breeding innovation of *Cymbidium ensifolium* var. susin accessions. This study provides important theoretical support for hybrid parent selection, molecular marker-assisted breeding and germplasm innovative utilization of *Cymbidium ensifolium*, and is of great significance for the conservation and genetic improvement of Chinese orchids.

## Figures and Tables

**Table 1:** Details of 13 germplasms of *Cymbidium ensifolium* var. susin.

No.	Variety	Abbreviation	Country of Origin
1	Qingshanyuquan	QSYQ	Nanjing County, Fujian Province, China
2	Bianchenggongsu	BCGS	Xiangxi Tujia and Miao Autonomous Prefecture, Hunan Province, China
3	Liuyangsu	LiuYS	Liuyang City, Hunan Province, China
4	Tiegusu	TGS	Puning City, Guangdong Province, China
5	Tiegujinqi	TGJQ	Puning City, Guangdong Province, China
6	Dafengsu	DFS	Nanjing County, Fujian Province, China
7	Dahesu	DHS	Nanjing County, Fujian Province, China
8	Renhuabai	RHB	Renhua County, Guangdong Province, China
9	Jinsimawei	JSMW	Nanjing County, Fujian Province, China
10	Longyansu	LongYS	Longyan City, Fujian Province, China
11	Yahuangsu	YHS	Nanjing County, Fujian Province, China
12	Yinbiandagong	YBDG	Meizhou City, Guangdong Province, China
13	Emeixue	EMX	EmeiShan City, Sichuan Province, China

**Table 2:** Methods for measurement of quantitative traits and coding rules for qualitative traits.

Traits	Abbreviation	Description
Plant height	PH	The height from the base of the leaf blade to the upper plane of the plant
Leaf number	LN	The number of leaves per plant
Leaf length	LeL	The longest leaf length in a single leaf
Leaf width	LeW	Width of the broadest part of the longest leaf of a plant
Scape with flowers	SWF	The number of individual scape flowers
Pedicel length	PedL	The length of a peduncle in which a single flower is attached to the main body of an inflorescence
Pedicel width	PedW	Pedicel diameter measured by vernier caliper
Flower length	FL	Mean value of flower length
Flower width	FW	Mean value of flower width
Median sepal length	MSL	Mean lengths of median sepals
Median sepal width	MSW	Mean width of median sepal
Lateral sepal length	LSL	Mean length of lateral sepals
Lateral sepal width	LSW	Mean width of lateral sepals
Petal length	PetL	Straight distance from base to tip of petal
Petal width	PetW	The distance between the sides of a petal
Lip length	LiL	The length of the labellum from base to tip when extended
Lip width	LiW	The distance between the sides when the labellum is extended
Number of Petal colors	PCN	Number of petal color types
Number of sepal colors	SCN	Number of sepal color types
Number of lip colors	LCN	Number of lip color types
Plant type	PT	Erect = 1, semi-erect = 2
Blade shape	BS	Lanceolate = 1, linear = 2, oblanceolate = 3
Leaf tip shape	LTS	Sharp = 1, Round = 2
Petal posture	PP	Full inflection, partial inflection = 1, Partially Flat = 2, Part Flat, part rewinding = 3, Part Rat Race, part rewind = 4
Median sepal shape	MSS	Linear = 1, oblong = 2, obovate = 3
Lateral sepal shape	LSS	Lanceolate = 1, linear = 2, oblong = 3
Petal shape	PS	Oblong = 1, Oval = 2
Lip shape	LS	Triangles = 1, trapezoid = 2
Twisted leaves	TL	No = 1, yes = 2
Leaf surface markings	LSM	No = 1, yes = 2

**Table 3:** Genetic diversity analysis of quantitative characters of *Cymbidium ensifolium* var. susin.

Trait	Mean	SD	Maximum	Minimum	CV (%)	Genetic Diversity Index (H′)
Plant height (PH)	39.01	2.94	45.43	34.54	7.54	1.84
Leaf number (LN)	4.10	0.58	5.00	3.33	14.25	1.70
Leaf length (LeL)	41.44	2.76	50.08	39.25	6.66	1.16
Leaf width (LeW)	1.52	0.18	1.80	1.14	12.19	1.67
Scape with flowers (SWF)	5.77	0.53	6.67	4.67	9.24	1.69
Pedicel length (PedL)	26.29	2.16	29.19	20.67	8.21	1.67
Pedicel width (PedW)	0.37	0.05	0.48	0.30	12.52	1.78
Flower length (FL)	4.61	0.35	5.15	3.90	7.59	1.93
Flower width (FW)	3.61	0.35	4.09	2.76	9.62	1.67
Median sepal length (MSL)	2.77	0.20	3.03	2.40	7.06	1.56
Median sepal width (MSW)	0.82	0.11	1.05	0.70	13.45	1.73
Lateral sepal length (LSL)	2.67	0.23	2.93	2.20	8.63	1.78
Lateral sepal width (LSW)	0.66	0.05	0.74	0.57	6.92	1.82
Petal length (PetL)	2.32	0.18	2.54	1.99	7.60	1.78
Petal width (PetW)	0.78	0.11	1.10	0.67	14.18	1.52
Lip length (LiL)	1.98	0.14	2.23	1.70	6.86	1.63
Lip width (LiW)	0.77	0.09	1.03	0.65	12.14	1.41
Number of Petal colors (PCN)	1.23	0.44	2	1	35.63	0.54
Number of sepal colors (SCN)	1.31	0.48	2	1	36.74	0.62
Number of labellum colors (LCN)	1.31	0.48	2	1	36.74	0.62

**Table 4:** Frequency distribution and diversity of quality traits.

Trait	Frequency Distribution (%)				H′
	1	2	3	4	
Plant type (PT)	23.08	76.92			0.54
Blade shape (BS)	69.23	7.69	23.08		0.79
Leaf tip shape (LTS)	61.54	38.46			0.67
Petal posture (PP)	30.77	23.08	38.46	7.69	1.27
Median sepal shape (MSS)	30.77	53.85	15.38		0.98
Lateral sepal shape (LSS)	7.69	84.62	7.69		0.54
Petal shape (PS)	76.92	23.08			0.54
Labial shape (LS)	38.46	61.54			0.67
Twisted leaves (TL)	76.92	23.08			0.54
Leaf surface markings (LSM)	30.77	69.23			0.62

**Table 5:** Principal component analysis of *Cymbidium ensifolium* var. susin.

Trait	PC1	PC2	PC3	PC4	PC5	PC6	PC7	PC8
Plant height (PH)	0.22	0.16	0.29	−0.01	−0.19	−0.07	0.09	−0.02
Leaf number (LN)	0.20	−0.24	0.05	0.10	0.25	0.10	0.11	0.19
Leaf length (LeL)	0.05	−0.06	0.38	0.22	−0.17	−0.08	0.15	0.09
Leaf width (LeW)	0.26	0.07	0.27	−0.19	−0.10	0.02	−0.03	0.02
Scape with flowers (SWF)	−0.25	0.08	0.00	−0.04	−0.37	0.05	0.04	−0.09
Pedicel length (PedL)	−0.07	0.08	0.12	0.27	−0.31	0.16	−0.01	0.44
Pedicel width (PedW)	0.16	−0.12	0.13	0.20	0.00	0.01	−0.51	−0.05
Flower length (FL)	0.26	0.17	0.25	−0.12	0.00	−0.02	−0.06	0.11
Flower width (FW)	0.18	0.15	0.12	−0.29	0.29	0.07	−0.02	0.17
Median sepal length (MSL)	0.31	−0.15	−0.02	0.19	−0.10	−0.01	0.06	0.18
Median sepal width (MSW)	0.02	0.33	−0.11	−0.10	−0.12	0.34	0.03	−0.01
Lateral sepal length (LSL)	0.29	0.06	−0.23	0.15	−0.01	0.12	0.05	0.06
Lateral sepal width (LSW)	0.05	0.13	−0.23	0.10	0.37	−0.24	0.17	0.15
Petal length (PetL)	0.27	0.12	−0.08	0.22	0.16	0.20	0.09	0.03
Petal width (PetW)	0.08	0.35	0.07	0.06	−0.08	−0.10	0.30	0.05
Lip length (LiL)	0.02	0.21	−0.32	0.13	−0.04	0.15	−0.13	0.04
Lip width (LiW)	−0.01	0.37	−0.04	−0.08	0.14	−0.18	0.09	0.12
Petal color type number (PCN)	−0.31	−0.09	−0.06	0.27	0.07	−0.01	−0.05	0.10
Sepal color type number (SCN)	0.16	−0.14	0.24	0.01	0.16	−0.15	0.11	−0.45
Lip color type number (LCN)	0.09	0.19	−0.11	0.36	0.00	0.00	−0.34	−0.15
Plant type (PT)	0.21	−0.02	−0.20	−0.08	−0.32	0.08	0.30	−0.06
Blade shape (BS)	−0.23	−0.07	0.23	0.05	0.17	0.23	0.26	−0.05
Leaf tip shape (LTS)	0.14	0.11	0.01	0.38	0.03	−0.25	0.07	−0.03
Petal posture (PP)	−0.03	0.21	0.24	0.14	0.28	0.22	−0.18	−0.17
Median sepal shape (MSS)	−0.18	0.11	0.24	0.07	0.22	0.26	0.05	0.20
Lateral sepal shape (LSS)	−0.05	0.30	−0.01	0.15	−0.05	−0.12	0.12	−0.49
Petal shape (PS)	−0.29	0.24	0.05	0.06	0.13	−0.03	0.13	0.05
Labial shape (LS)	−0.10	0.22	0.25	0.01	−0.16	−0.10	−0.27	0.06
Twisted leaves (TL)	0.11	0.00	−0.02	−0.03	0.01	0.59	0.03	−0.26
Leaf surface markings (LSM)	−0.06	−0.18	0.11	0.36	−0.05	0.12	0.32	−0.09
eigenvalue	7.01	5.57	4.60	2.99	2.86	2.00	1.84	1.29
Percentage of variance (%)	23.38	18.58	15.34	9.95	9.54	6.67	6.12	4.30
cumulative (%)	23.38	41.96	57.30	67.25	76.79	83.46	89.59	93.89

**Table 6:** Statistics of data quality control results and comparison results of *Cymbidium ensifolium* var. susin.

Sample	Raw Reads	Clean Reads	Q20_Ratio	Q30_Ratio	CG Content	Mapped Reads	Mapped Rate	Depth
DHS	13,336,686	13,329,160	96.09%	90.31%	39.25%	13,243,509	99.36%	0.47
DFS	13,241,744	13,234,370	95.73%	89.43%	40.26%	13,123,249	99.16%	0.46
TGS	15,341,026	15,332,440	95.81%	89.63%	39.58%	15,190,602	99.07%	0.53
LongYS	15,845,632	15,836,956	96.00%	90.12%	38.85%	15,712,751	99.22%	0.56
LiuYS	15,172,078	15,163,276	96.14%	90.34%	39.79%	15,026,392	99.10%	0.53
RHB	17,186,652	17,176,852	96.34%	90.89%	40.03%	17,046,800	99.24%	0.60
JSMW	16,056,198	16,047,398	96.30%	90.72%	40.76%	15,916,622	99.19%	0.57
BCGS	19,013,762	19,003,850	95.52%	88.87%	39.05%	18,836,851	99.12%	0.67
YBDG	9,414,972	9,409,488	96.22%	90.60%	39.37%	9,320,624	99.06%	0.32
TGJQ	22,337,560	22,324,604	96.24%	90.54%	39.38%	22,078,031	98.90%	0.77
EMX	13,855,316	13,847,858	95.84%	89.72%	39.27%	13,754,275	99.32%	0.49
QSYQ	15,768,954	15,759,996	96.28%	90.73%	39.82%	15,641,243	99.25%	0.55
YHS	19,253,196	19,242,736	95.53%	89.06%	38.67%	19,102,511	99.27%	0.68

**Table 7:** Genetic diversity of core and reserved collections of *Cymbidium ensifolium* var. susin.

Sampling	Sample. Population	Sample. Numbers	Number of Polymorphic Loci	Polymorphic Loci Numbers (%)	Ne	H′	He
25	core germplasm	1	19,063	31.72	1.33 ± 0.47	0.33 ± 0.47	0.25 ± 0.35
25	non-core germplasm	1	17,875	29.74	1.33 ± 0.46	0.32 ± 0.46	0.24 ± 0.35
50	core germplasm	3	39,456	65.65	1.45 ± 0.38	0.55 ± 0.41	0.46 ± 0.33
50	non-core germplasm	3	32,019	53.27	1.39 ± 0.41	0.46 ± 0.44	0.38 ± 0.35
75	core germplasm	4	48,417	80.55	1.49 ± 0.34	0.63 ± 0.35	0.55 ± 0.27
75	non-core germplasm	5	46,459	77.3	1.45 ± 0.35	0.58 ± 0.36	0.52 ± 0.29
100	core germplasm	6	55,184	91.81	1.51 ± 0.30	0.68 ± 0.27	0.61 ± 0.19
100	non-core germplasm	7	49,173	81.81	1.45 ± 0.35	0.58 ± 0.35	0.54 ± 0.26

## Data Availability

Sequence quality control and filtering were performed using fastp v0.23.4. Sequence alignment was conducted with BWA v0.7.17. Variant calling and filtering were implemented using GATK v4.4.0.0. Variant annotation was carried out with ANNOVAR v20200607 and VCFtools v0.1.17, respectively. Phylogenetic tree construction was performed using PHYLIP v3.696. Population structure analysis (PCA) and ancestry component analysis were conducted using GCTA v1.93.2 and ADMIXTURE v1.3, respectively. Core germplasm analysis was performed using GenoCore (latest version). All software tools are publicly available at their respective official websites.
